# Deaths in custody in Senegal from 2017-2019: a retrospective chart review

**DOI:** 10.1108/IJOPH-10-2023-0068

**Published:** 2024-09-02

**Authors:** Famara Seck, Stuart A. Kinner, Rohan Borschmann

**Affiliations:** Department of Forensic Science, Penitentiary Administration, Dakar, Senegal; Centre for Adolescent Health, Murdoch Childrens Research Institute, Parkville, Australia and School of Population and Global Health, The University of Melbourne, Parkville, Australia; Centre for Mental Health, The University of Melbourne School of Population and Global Health, Melbourne, Australia and Department of Psychiatry, Oxford University, Oxford, UK

**Keywords:** Incarceration, Mortality, Senegal, Prison, Cardiovascular disease, Cancer, Infectious diseases, Suicide

## Abstract

**Purpose:**

This study aims to document the incidence and causes of deaths in custody in Senegal from 2017 to 2019 and to describe the demographic and criminal justice characteristics of decedents.

**Design/methodology/approach:**

The authors examined medical records and death reports relating to all deaths occurring between January 1, 2017 and December 31, 2019 during a period of incarceration in Senegal.

**Findings:**

Among the estimated 83,568 people incarcerated in Senegal during the study period, 83 deaths were recorded in custody; 24 in 2017, 32 in 2018 and 27 in 2019. This resulted in a rate of 1.0 deaths per 1,000 incarcerated people. Of the 83 decedents identified, 79 (95%) were males. Similar proportions of decedents were serving custodial sentences (*n* = 44; 53%) and awaiting trial (*n* = 39; 47%) at the time of death. Most deaths were recorded as being because of natural causes (*n* = 67; 81%); the most common causes recorded were cardiovascular disease (*n* = 22; 27%), cancer (*n* = 12; 15%) and infectious diseases (*n* = 11; 13%). Two people (2.4%) died by suicide, and one (1.2%) died as a result of interpersonal violence. Most deaths (*n* = 59; 71%) occurred in hospitals, 14 (17%) occurred in prisons and 7 (8%) occurred in prison health centers.

**Originality/value:**

The authors observed a higher rate of death and a markedly lower proportion of deaths in custody in Senegal because of suicide and violence, when compared with similar studies from high-income countries. The findings of this study point to a need for greater investment in screening, health care and health promotion in custodial settings to reduce potentially preventable deaths among people in custody in Senegal.

## Introduction

The global prison population has increased by 24% since 2000 (Walmsley, 2018), and the United Nations (UN) estimates that more than 30 million people are released from prisons globally each year (UNODC, 2008). The criminal justice system functions as a filter for marginalized, unwell and under-served members of the community (Marmot, 2018) – a process that has been described as the “sedimentation of disease” (Ross, 2012). People who experience incarceration are distinguished by remarkably poor health profiles, including elevated rates of mental illness (Fazel and Danesh, 2002), substance use disorders (Cutcher *et al.*, 2014; Milloy *et al.*, 2009), infectious and non-communicable diseases (NCDs) (AIHW, 2019) and cognitive disability (Fazel and Baillargeon, 2011; Dolan *et al.*, 2016; Kinner and Young, 2018; Borschmann *et al.*, 2020). These complex, co-occurring health problems often interact in a syndemic fashion (Culbert *et al.*, 2016) and occur against a backdrop of trauma, abuse and entrenched intergenerational disadvantage (Hughes *et al.*, 2020; Pettit and Western, 2004; Borschmann, 2019). These poor health profiles contribute to a mortality risk that is markedly higher than that of their age- and sex-matched peers in the general population (Binswanger *et al.*, 2007; Kinner *et al.*, 2013; Spittal *et al.*, 2014). Most previous research on deaths in custody has been conducted in high-income countries including Australia (Dalton, 1999; Austin *et al.*, 2014; Larney *et al.*, 2014), the UK (van Ginneken *et al.*, 2017) and the USA (Binswanger *et al.*, 2014; Noonan and Ginder, 2015; Rosen *et al.*, 2011), with many of these studies focusing on deaths because of suicide (Fazel *et al.*, 2017; Zhong *et al.*, 2021; Fazel *et al.*, 2011; Austin *et al.*, 2014; van Ginneken *et al.*, 2017) or drug overdose (Kinner *et al.*, 2013; Bird and Hutchinson, 2003). This focus appears warranted, in light of the high proportion of deaths because of suicide and overdose after people are released from incarceration in these countries (Binswanger *et al.*, 2007; Binswanger *et al.*, 2013; Borschmann *et al.*, 2024).

Previous prison research in Africa has documented overcrowded and unsanitary living conditions, high rates of infectious diseases and an increased risk of mortality in several countries including Tanzania (Rutta *et al.*, 2001), Ethiopia (Tirfeneh *et al.*, 2017), Burkina Faso (Diendéré *et al.*, 2021), South Africa (Jansen and Tendayi Achiume, 2011; Pillay and Aldous, 2019), the Democratic Republic of Congo (Kalonji *et al.*, 2019), Cote d’Ivoire (Anongba and Adeoti, 2013), Malawi (Nyangulu *et al.*, 1997; Van Hout *et al.*, 2022b), Tunisia (Jedidi *et al.*, 2018) and Zimbabwe (Alexander, 2009; Mukwenha *et al.*, 2021; Van Hout *et al.*, 2022a). The disproportionately high rate of tuberculosis (TB) documented in several prisons in sub-Saharan Africa has been referred to as a “potential time bomb” (O'Grady *et al.*, 2011), particularly given that approximately 70% of incarcerated people with TB also live with human immunodeficiency virus (HIV). Yet prisons often have inadequate resources to treat either infection (O'Grady *et al.*, 2011), and access to health care among incarcerated people is often inadequate (Nweze *et al.*, 2021). One study of prisons in 18 sub-Saharan African countries (Van Hout *et al.*, 2018) documented “appalling prison health care provision” and noted that compromised access to health-care services exacerbated the spread of infectious diseases such as HIV infection and TB.

Although prison health research internationally has focused disproportionately on HIV and TB, the prevalence of NCDs among people exposed to the criminal justice system is also high in many countries (PRI, 2022). A recent study of 48,670 young people (aged 10–18 years) charged with a criminal offence in Queensland, Australia reported that one in every 12 deaths was because of NCDs and that the rate of death because of NCDs was more than 1.5 times higher than among their age- and sex-matched peers who had not been charged with any offences (Calais-Ferreira *et al.*, 2023). A global systematic review of NCD risk factors in prisons (Herbert *et al.*, 2012) identified 29 publications involving >60,000 incarcerated adults across 15 countries and called for both further research and increased monitoring to drive meaningful reform. The review included six studies from Cameroon, Nigeria, Ivory Coast, East and West Africa, identifying issues of malnourishment and dietary deficiencies. Notably, none of the included studies reported on the prevalence of NCDs. Despite this, the health of people incarcerated in sub-Saharan Africa is, by and large, a neglected political and research issue (O’Grady *et al.*, 2011).

Rule 24.1 of the UN Standard Minimum Rules for the Treatment of Prisoners (the “Nelson Mandela Rules” [UNODC, 2015]) states that incarcerated people “should enjoy the same standards of health care that are available in the community and should have access to necessary health-care services free of charge without discrimination on the grounds of their legal status” (the “principle of equivalence”; p.12). However, multiple studies have documented inadequate prison health care in sub-Saharan Africa (Van Hout and Mhlanga-Gunda, 2019; UN, 2018), including in the West African country of Senegal (Paul *et al.*, 2020). Despite this, and the high prevalence of disease in Senegalese prisons (Paul *et al.*, 2020), little is known about deaths in prisons in this country. In this study, using prison medical reports and death records, we aimed to estimate the incidence of all-cause and cause-specific mortality occurring in prisons in Senegal between January 1, 2017 and December 31, 2019 and to examine the demographic and criminogenic characteristics of decedents.

## Methodology

### Study design and setting

We conducted a retrospective chart review of all deaths in custody (including prisons, prison health centers, external hospitals while incarcerated and in transit between these) occurring between January 1, 2017 and December 31, 2019 in Senegal. More than 27,000 people are incarcerated each year in Senegal, across 37 custodial institutions. Of these institutions, 32 are short-term correctional centers which accommodate people sentenced to a term of incarceration from 1 to 15 days.

### Inclusion and exclusion criteria

We included any death of an incarcerated person which occurred between January 1, 2017 and December 31, 2019 either in prison; in a hospital or health center while in custody; or during transit between prison and a hospital. There were no exclusion criteria.

### Data collection and analysis

A senior, experienced physician (FS) reviewed the medical records and death reports relating to any death of an incarcerated person meeting the inclusion criteria listed above. The following data were manually extracted from the medical records and death reports relating to all decedents: age at death, nationality, sex, conviction status (i.e. convicted *vs* awaiting trial), date and place of hospitalization, date of death, cause(s) of death (using International Classification of Disease – 10th edition [ICD-10] [WHO, 1992] codes for underlying cause of death) and place of death.

## Results

An estimated total of 83,568 people were incarcerated during the study period (26,022 in 2017; 27,720 in 2018; and 29,826 in 2019). A total of 83 deaths were recorded during an episode of incarceration: 24 in 2017, 32 in 2018 and 27 in 2019. Based on these figures, the rate of all-cause death among incarcerated people in Senegal was 1.0 deaths per 1,000 people.

### Demographic characteristics of decedents

Of the 83 decedents, 79 (95%) were males, the median age at death was 42 years (range: 17–87 years; and IQR: 33–53 years) and most (*n* = 74, 89%) were Senegalese. In all, 44 decedents (53%) were serving custodial sentences and 39 (47%) were awaiting trial. The demographic and criminogenic profiles of decedents in our study broadly matched the profiles of the wider population of people incarcerated in Senegal at the time the study was conducted.

### Causes and location of death

The cause of death was listed as natural causes in 67 (81%) cases. The most common causes were cardiovascular diseases (*n* = 22; 27%), cancer (*n* = 12; 14%) and infectious diseases (*n* = 11; 13%). Two people (2%) died by suicide, two (2%) died as the result of an accident and one person (1%) died as a result of interpersonal violence. The cause of death was listed as “unknown” in 11 (13%) cases. Most deaths occurred in people aged 20–39 years (31; 37%) or 40–59 years (34; 41%). Two in five deaths (*n* = 20/49, 41%) among those aged 40 years or older were attributed to cardiovascular disease. Most decedents died in either a hospital (*n* = 59; 71%) or a prison health center (7; 8%), while 14 (17%) died elsewhere within the prison complex.

## Discussion

We aimed to estimate the incidence of deaths in custody in Senegal from 2017 to 2019 and to describe the demographic and criminogenic characteristics of decedents. In all, 83 deaths were recorded during the study period, resulting in a conservatively estimated rate of 1.0 deaths for every 1,000 people incarcerated in Senegal. Cardiovascular disease, cancer and infectious diseases – the three most common causes of death – accounted for 54% of all recorded deaths. Most decedents were male, and most deaths occurred in hospitals. Most decedents were aged between 20 and 59 years, which is markedly lower than the Senegalese population life expectancy of 69 years from 2019 when the study was conducted (WorldBank, 2023).

Compared with similar studies from high-income countries, our findings from Senegal, a low-income country in West Africa, revealed a higher proportion of deaths because of cardiovascular disease (Wobeser *et al.*, 2002) and lower proportions of death because of suicide (Fazel *et al.*, 2017) and violence (Brittain *et al.*, 2013; El Khal *et al.*, 2019; Désesquelles *et al.*, 2018). We also documented a markedly lower proportion of deaths because of suicide (Gowda *et al.*, 2021) and infectious diseases (Sonar, 2010) than in previous research from other low-income countries. Our conservatively estimated rate of 1.0 deaths per 1,000 incarcerated people in Senegal is three to four times higher than recent data from the USA, which reported a rate of 0.26 deaths per 1,000 people in federal prisons and 0.33 deaths per 1,000 people in state prisons (Carson, 2021). Our findings suggest a substantial burden of chronic NCDs among people who experience incarceration in Senegal and point to a need for greater investment in health promotion, prison medical care and initiatives to mitigate structural inequities in health status and care, to reduce the burden of NCD mortality in this highly marginalized segment of the Senegalese population. The low proportion of deaths because of suicide we observed (2%) warrants further investigation, particularly in relation to identifying risk and protective factors distinct from those identified in research from high-income countries (Fazel *et al.*, 2017). Further research on the health status of people in Senegalese prisons, and on prison health care in Senegal, will be required to inform and target such investments. Rule 24.1 of the Mandela Rules (UNODC, 2015) requires that standards of health care in custody should be the same as in the surrounding community. However, in light of evidence that health care in the Senegalese community is sub-optimal, inequitable and fragmented (Paul *et al.*, 2020), community-equivalence may not be an adequate standard for prison health care in Senegal (Lines, 2006). Disproportionate investment in prison health care will be required to mitigate the health inequities experienced by people who experience incarceration in Senegal.

### Strengths and limitations

To our knowledge, these are the first published data resulting from a rigorous examination of deaths occurring in custody in Senegal and among the first from any country in Africa (Alexander, 2009; Jedidi *et al.*, 2018; Kalonji *et al.*, 2019; Pillay and Aldous, 2019). Further work, including in other countries, with larger samples and with richer information on the medical and other characteristics of decedents, should be considered a priority for future researchers. Review of both medical records and death reports allowed us to describe the demographic and criminogenic characteristics of decedents and to characterize causes of death. Our study had four main limitations. First, because of limitations of Senegalese prison records, we were unable to identify individuals who experienced incarceration across multiple calendar years during the study period. As such, our estimate of the number of unique individuals incarcerated during the study period is likely inflated. Our estimate of the mortality rate in Senegalese prisons is, accordingly, conservative. Second, one in every eight deaths resulted from “unknown causes” and drawing policy-relevant conclusions about these deaths is, therefore, difficult. This limitation likely relates to the completeness of cause of death data in Senegal, including deaths occurring during an episode of incarceration. While it is certain that the rate of death is higher than in many high-income countries, further work is required to definitively establish the causes of death and to inform meaningful prevention efforts. Third, we did not examine deaths after incarceration. However, we recently documented a high rate of deaths after incarceration in multiple countries (Borschmann *et al.*, 2024). In this study, we noted a lack of data from low- and middle-income countries, where the burden of NCDs predominates at the population level; as such, efforts to examine deaths after incarceration in Senegal and other sub-Saharan African countries are urgently required. Finally, our findings were based exclusively on the information contained within medical records and death reports, and the accuracy of such information has not been systematically examined. Recent evidence from mortality data across 20 countries has documented an inverse relationship between a country’s socio-demographic development and the overall quality of the country’s cause of death data (Iburg *et al.*, 2020). Data on deaths in prisons – which, in many ways, are a sentinel site for public health surveillance – have public health utility only to the extent that they are accurate. Further work to review the quality of death data in prisons in Senegal and other sub-Saharan African countries may reveal opportunities to enhance their utility.

### Conclusions and recommendations

Almost all deaths occurring in custody in Senegal are attributable to either non-communicable or infectious diseases, with cardiovascular disease and cancer the two most common causes. Our findings reinforce the importance of effective, sustained health promotion and prevention initiatives that are inclusive of people who experience incarceration in Senegal. These initiatives should ideally focus on sustained efforts to improve health and reduce the onset, progression and severity of chronic and infectious diseases (e.g. improving diet, reducing substance use and increasing physical activity) both during and after episodes of incarceration. Investing in high-quality nutrition in accordance with World Health Organization (WHO) guidelines (WHO, 2022; Enggist *et al.*, 2014) would likely function as a critical and cost-effective mechanism for the prevention, management and potential reversal of many NCDs and could potentially also provide a platform for training incarcerated people in employable skills (e.g. food preparation and hygiene). Our findings point to the need for greater investment in screening, health care and health promotion in custodial settings to reduce potentially preventable deaths among people incarcerated in Senegal. To the extent that our findings reflect common health concerns among the most marginalized segments of Senegalese society, they also highlight opportunities for greater investment in equity-focused disease prevention, which may simultaneously improve health outcomes, reduce health inequalities and potentially reduce the incarceration of medically vulnerable individuals. Efforts to achieve sustained improvements in health trajectories after release from incarceration will likely yield measurable benefits at the population level, particularly in terms of health equity (Kinner and Wang, 2014).

Previous research on the health of people incarcerated in Africa has focused on infectious diseases (Telisinghe *et al.*, 2016), particularly HIV and TB. While this is undoubtedly important, our findings point to the need for greater investment in preventing and treating NCDs in this setting. NCD deaths in custody are indicative of health inequities in the wider Senegalese community; as many NCDs develop over a lifetime (and not merely during the relatively brief period spent in custody), it is likely that NCD deaths occurring in custody represent only a small fraction of the burden of preventable NCD deaths in Senegal. This NCD burden likely manifests in substantial public health and health-care costs, and individuals released from prison experience may experience rapid decompensation of disease. As such, routine monitoring of the health of people incarcerated in Senegal – and, ideally, of those recently released from incarceration – is important to informing efforts to improve health, health care and – in particular – health equity, at the population level. In efforts to progress toward the SDGs in African countries, including universal health care, people in prisons must not be left behind (Winkelman *et al.*, 2022; Paul *et al.*, 2020). Prisons should not be excluded from broader efforts to improve universal health care and health-care equity in Senegal.

## References

[ref001] AIHW (2019), The Health of Australia's Prisoners 2018, Australian Institute of Health and Welfare, Canberra.

[ref002] Alexander, J. (2009), “Death and disease in Zimbabwe's prisons”, The Lancet, Vol. 373 No. 9668, pp. 995-996. 10.1016/s0140-6736(09)60592-4 19330896

[ref003] Anongba, S. and Adeoti, M. (2013), “Prison health environment and patients safety in cote d'Ivoire”, Antimicrobial Resistance & Infection Control, Vol. 2, p. 1.23305311

[ref004] Austin, A.E., VAN DEN Heuvel, C. and Byard, R.W. (2014), “Prison suicides in South Australia: 1996–2010”, Journal of Forensic Sciences, Vol. 59 No. 5, pp. 1260-1262. 24635128 10.1111/1556-4029.12454

[ref005] Binswanger, I.A., Blatchford, P.J., Mueller, S.R. and Stern, M.F. (2013), “Mortality after prison release: opioid overdose and other causes of death, risk factors, and time trends from 1999 to 2009”, Annals of Internal Medicine, Vol. 159 No. 9, pp. 592-600. 24189594 10.7326/0003-4819-159-9-201311050-00005 PMC5242316

[ref006] Binswanger, I.A., Carson, E.A., Krueger, P.M., Mueller, S.R., Steiner, J.F. and Sabol, W.J.J.B. (2014), “Prison tobacco control policies and deaths from smoking in United States prisons: population based retrospective analysis”, p. 349. 10.1136/bmj.g4542 PMC4122735 25097186

[ref007] Binswanger, I.A., Stern, M.F., Deyo, R.A., Heagerty, P.J., Cheadle, A., Elmore, J.G. and Koepsell, T.D. (2007), “Release from prison – a high risk of death for former inmates”, New England Journal of Medicine, Vol. 356 No. 2, pp. 157-165. 17215533 10.1056/NEJMsa064115 PMC2836121

[ref008] Bird, S.M. and Hutchinson, S.J. (2003), “Male drugs‐related deaths in the fortnight after release from prison: Scotland, 1996–99”, Addiction (Abingdon, England), Vol. 98 No. 2, pp. 185-190. 12534423 10.1046/j.1360-0443.2003.00264.x

[ref009] Borschmann, R. (2019), The Health of Adolescents in Detention: A Global Scoping Review, The Lancet Public Health. 10.1016/S2468-2667(19)30217-8 PMC7025881 31954434

[ref010] Borschmann, R., Janca, E., Willoughby, M., Fazel, S., Hughes, N., Patton, G.C., Sawyer, S.M., Love, A., Puljević, C., Stockings, E., Hill, N., Hocking, J., Robinson, J., Snow, K., Carter, A. and Kinner, S.A. (2020), “The health of adolescents in detention: a global scoping review”, The Lancet Public Health, Vol. 5 No. 2, pp. 114-126. 10.1016/S2468-2667(19)30217-8 PMC7025881 31954434

[ref011] Borschmann, R., Keen, C., Spittal, M.J., Preen, D., Pirkis, J., Larney, S., Rosen, D.L., Møller, L., O'moore, E. and Young, J.T. (2024), “Rates and causes of death after release from incarceration among 1 471 526 people in eight high-income and middle-income countries: an individual participant data meta-analysis”, The Lancet, Vol. 403 No. 10438, pp. 1779-1788. 10.1016/S0140-6736(24)00344-1 38614112

[ref012] Brittain, J., Axelrod, G. and Venters, H. (2013), “Deaths in New York city jails, 2001–2009”, American Journal of Public Health, Vol. 103 No. 4, pp. 638-640. 23409900 10.2105/AJPH.2012.301042 PMC3673256

[ref013] Calais-Ferreira, L., Young, J.T., Francis, K., Willoughby, M., Pearce, L., Clough, A., Spittal, M.J., Brown, A., Borschmann, R., Sawyer, S.M. and Kinner, S. (2023), “Non-communicable disease mortality in young people with a history of contact with the youth justice system in Queensland, Australia: a retrospective, population-based cohort study”, The Lancet Public Health, Vol. 8 No. 8, pp. e600-e609. 37516476 10.1016/S2468-2667(23)00144-5

[ref014] Carson, E. (2021), “Mortality in state and federal prisons, 2001–2019 – statistical tables”, Bureau of Justice Statistics, USA, available at: https://bjs.ojp.gov/library/publications/mortality-state-and-federal-prisons-2001-2019-statistical-tables

[ref015] Culbert, G.J., Pillai, V., Bick, J., Al-Darraji, H.A., Wickersham, J.A., Wegman, M.P., Bazazi, A.R., Ferro, E., Copenhaver, M., Kamarulzaman, A. and Altice, F.L. (2016), “Confronting the HIV, tuberculosis, addiction, and incarceration syndemic in southeast Asia: lessons learned from Malaysia”, Journal of Neuroimmune Pharmacology, Vol. 11 No. 3, pp. 446-455. 27216260 10.1007/s11481-016-9676-7 PMC5118227

[ref016] Cutcher, Z., Degenhardt, L., Alati, R. and Kinner, S.A. (2014), “Poor health and social outcomes for ex-prisoners with a history of mental disorder: a longitudinal study”, Australian and New Zealand Journal of Public Health, Vol. 38 No. 5, pp. 424-429. 24962322 10.1111/1753-6405.12207

[ref017] Dalton, V. (1999), “Death and dying in prison in Australia: national overview, 1980–1998”, Journal of Law, Medicine & Ethics, Vol. 27 No. 3, pp. 269-274. 10.1111/j.1748-720x.1999.tb01461.x 11067604

[ref018] Désesquelles, A., Kensey, A., Meslé, F. and VAN Hoorn Alkema, B.J.P. (2018), “Circumstances and causes of death among prisoners in France: the preponderance of violent deaths”, Vol. 73, pp. 721-750.

[ref019] Diendéré, E.A., Traoré, K., Bernatas, J.-J., Idogo, O., Dao, A.K., Traoré, G.K., Napon, P.D., Ouédraogo, S., Bognounou, R. and Diallo, I. (2021), “Prison health priorities in Burkina Faso: a cross-sectional study in the two largest detention environments in Burkina Faso”, International Journal of Prisoner Health, Vol. 18 No. 1. 10.1108/IJPH-04-2021-0036 34392661

[ref020] Dolan, K., Wirtz, A., Moazen, B., Galvani, A., Ndeffo-Mbah, M., Kinner, S., Courtney, R., Mckee, M., Amon, J.J., Hellard, M., Altice, F. and Beyrer, C. (2016), “Global burden of HIV, viral hepatitis, and tuberculosis in prisoners and detainees”, The Lancet, Vol. 388 No. 10049, pp. 1089-1102. 10.1016/S0140-6736(16)30466-4 27427453

[ref021] EL Khal, M.C., Grill, S., Costagliola, R., Blanc, A., Savall, F. and Telmon, N. (2019), “Deaths in custody in the Toulouse region – autopsy study between 2011 and 2017”, Med Leg Rev, Vol. 10, pp. 57-62.

[ref022] Enggist, S., Møller, L., Galea, G. and Udesen, C. (2014), “Prisons and health”, World Health Organization, Regional Office for Europe.

[ref023] Fazel, S. and Baillargeon, J. (2011), “The health of prisoners”, The Lancet, Vol. 377 No. 9769, pp. 956-965. 10.1016/S0140-6736(10)61053-7 21093904

[ref024] Fazel, S. and Danesh, J. (2002), “Serious mental disorder in 23 000 prisoners: a systematic review of 62 surveys”, The Lancet, Vol. 359 No. 9306, pp. 545-550. 10.1016/S0140-6736(02)07740-1 11867106

[ref025] Fazel, S., Grann, M., Kling, B., Hawton, K.J.S.P. and Epidemiology, P. (2011), “Prison suicide in 12 countries: an ecological study of 861 suicides during 2003–2007”, Vol. 46, pp. 191-195. 10.1007/s00127-010-0184-4 20140663

[ref026] Fazel, S., Ramesh, T. and Hawton, K. (2017), “Suicide in prisons: an international study of prevalence and contributory factors”, The Lancet Psychiatry, Vol. 4 No. 12, pp. 946-952. 29179937 10.1016/S2215-0366(17)30430-3 PMC6066090

[ref027] Gowda, G.S., Shadakshari, D., Vajawat, B., Reddi, V.S.K., Math, S.B. and Murthy, P. (2021), “Suicide in Indian prisons”, The Lancet Psychiatry, Vol. 8 No. 8, p. e19. 34303410 10.1016/S2215-0366(21)00233-9

[ref028] Herbert, K., Plugge, E., Foster, C. and Doll, H. (2012), “Prevalence of risk factors for non-communicable diseases in prison populations worldwide: a systematic review”, The Lancet, Vol. 379 No. 9830, pp. 1975-1982. 10.1016/S0140-6736(12)60319-5 22521520

[ref029] Hughes, N., Ungar, M., Fagan, A., Murray, J., Atilola, O., Nichols, K., Garcia, J. and Kinner, S. (2020), “The health determinants of adolescent criminalisation”, The Lancet Child & Adolescent Health, Vol. 4 No. 2, pp. 151-162. 31956016 10.1016/S2352-4642(19)30347-5

[ref030] Iburg, K.M., Mikkelsen, L., Adair, T. and Lopez, A.D. (2020), “Are cause of death data fit for purpose? Evidence from 20 countries at different levels of socio-economic development”, Plos One, Vol. 15 No. 8, p. e0237539. 32834006 10.1371/journal.pone.0237539 PMC7446871

[ref031] Jansen, R. and Tendayi Achiume, E. (2011), “Prison conditions in South Africa and the role of public interest litigation since 1994”, South African Journal on Human Rights, Vol. 27 No. 1, pp. 183-191.

[ref032] Jedidi, M., Mlayeh, S., Mahjoub, M., Mezgar, Z., Masmoudi, T., Dhiab, M.B., Zemni, M. and Souguir, M.K. (2018), “Death in detention in Sousse, Tunisia: a 10-year autopsy study”, Egyptian Journal of Forensic Sciences, Vol. 8 No. 1, pp. 1-4.

[ref033] Kalonji, G.M.P., Okenge, L.N., Ilunga-Ilunga, F., Albert, A. and Giet, D. (2019), “Factors associated with prison survival: study in the democratic republic of Congo”, Sante Publique (Vandoeuvre-les-Nancy, France), Vol. 31 No. 5, pp. 715-722. 32372610 10.3917/spub.195.0715

[ref034] Kinner, S.A. and Wang, E.A. (2014), “The case for improving the health of ex-prisoners”, American Journal of Public Health, Vol. 104 No. 8, pp. 1352-1355. 24922122 10.2105/AJPH.2014.301883 PMC4103236

[ref035] Kinner, S.A. and Young, J.T. (2018), “Understanding and improving the health of people who experience incarceration: an overview and synthesis”, Epidemiologic Reviews, Vol. 40 No. 1, pp. 4-11. 29860342 10.1093/epirev/mxx018 PMC5982728

[ref036] Kinner, S.A., Forsyth, S. and Williams, G. (2013), “Systematic review of record linkage studies of mortality in ex‐prisoners: why (good) methods matter”, Addiction, Vol. 108 No. 1, pp. 38-49. 23163705 10.1111/add.12010

[ref037] Larney, S., Gisev, N., Farrell, M., Dobbins, T., Burns, L., Gibson, A., Kimber, J. and Degenhardt, L. (2014), “Opioid substitution therapy as a strategy to reduce deaths in prison: retrospective cohort study”, BMJ Open, Vol. 4 No. 4, p. e004666. 10.1136/bmjopen-2013-004666 PMC3987723 24694626

[ref038] Lines, R. (2006), “From equivalence of standards to equivalence of objectives: the entitlement of prisoners to health care standards higher than those outside prisons”, International Journal of Prisoner Health, Vol. 2 No. 4, pp. 269-280.

[ref039] Marmot, M. (2018), “Inclusion health: addressing the causes of the causes”, The Lancet, Vol. 391 No. 10117, pp. 186-188. 10.1016/S0140-6736(17)32848-9 29137870

[ref040] Milloy, M.S., Buxton, J., Wood, E., Li, K., Montaner, J.S. and Kerr, T. (2009), “Elevated HIV risk behaviour among recently incarcerated injection drug users in a Canadian setting: a longitudinal analysis”, BMC Public Health, Vol. 9 No. 1, p. 156. 19473508 10.1186/1471-2458-9-156 PMC2695456

[ref041] Mukwenha, S., Dzinamarira, T., Mapingure, M.P. and Musuka, G. (2021), “Zimbabwe’s prison facilities: preparedness for institutional COVID-19 outbreaks”, Public Health in Practice, Vol. 2, p. 100089. 33564751 10.1016/j.puhip.2021.100089 PMC7862897

[ref042] Noonan, M.E. and Ginder, S. (2015), “Understanding mortality in state prison: do male prisoners have an elevated risk of death?”, Justice Research and Policy, Vol. 16 No. 1, pp. 65-80.

[ref043] Nweze, V.N., Anosike, U.G., Ogunwusi, J.F., Adebisi, Y.A. and Lucero-Prisno, D.E. III. (2021), “Prison health during the covid-19 era in Africa”, Public Health in Practice, Vol. 2, p. 100083. 33521740 10.1016/j.puhip.2021.100083 PMC7826114

[ref044] Nyangulu, D., Harries, A., Kang'ombe, C., Yadidi, A., Chokani, K., Cullinan, T., Maher, D., Nunn, P. and Salaniponi, F. (1997), “Tuberculosis in a prison population in Malawi”, The Lancet, Vol. 350 No. 9087, pp. 1284-1287. 10.1016/s0140-6736(97)05023-x 9357408

[ref045] O’grady, J., Hoelscher, M., Atun, R., Bates, M., Mwaba, P., Kapata, N., Ferrara, G., Maeurer, M. and Zumla, A. (2011), “Tuberculosis in prisons in Sub-Saharan Africa–the need for improved health services, surveillance and control”, Tuberculosis, Vol. 91 No. 2, pp. 173-178. 21251881 10.1016/j.tube.2010.12.002

[ref046] O'grady, J., Mwaba, P., Bates, M., Kapata, N. and Zumla, A. (2011), “Tuberculosis in prisons in Sub-Saharan Africa–a potential time bomb”, South African Medical Journal, Vol. 101 No. 2, pp. 107-108. 21678734 10.7196/samj.4629

[ref047] Paul, E., Ndiaye, Y., Sall, F.L., Fecher, F. and Porignon, D.J.H.P.O. (2020), “An assessment of the core capacities of the Senegalese health system to deliver universal health coverage”, Health Policy OPEN, Vol. 1, p. 100012. 32905018 10.1016/j.hpopen.2020.100012 PMC7462834

[ref048] Pettit, B. and Western, B. (2004), “Mass imprisonment and the life course: race and class inequality in U.S. incarceration”, American Sociological Review, Vol. 69 No. 2, pp. 151-169.

[ref049] Pillay, T. and Aldous, C.J.A.C.A.J.O.C. (2019), “Deaths in police custody in Durban, South Africa: 2004-2014”, Acta Criminologica: African Journal of Criminology Victimology, Vol. 32, pp. 129-143.

[ref050] PRI (2022), “Deaths in prison: examining causes, responses, and prevention of deaths in prison worldwide”, Penal Reform International, available at: https://cdn.penalreform.org/wp-content/uploads/2022/12/Deaths-in-prison-briefing.pdf

[ref051] Rosen, D.L., Wohl, D.A. and Schoenbach, V. (2011), “All-cause and cause-specific mortality among black and white North Carolina state prisoners, 1995–2005”, Annals of Epidemiology, Vol. 21 No. 10, pp. 719-726. 21737304 10.1016/j.annepidem.2011.04.007 PMC3166394

[ref052] Ross, M. (2012), Health and Health Promotion in Prisons, Routledge.

[ref053] Rutta, E., Mutasingwa, D., Ngallaba, S. and Mwansasu, A. (2001), “Tuberculosis in a prison population in Mwanza, Tanzania (1994–1997)”, The International Journal of Tuberculosis and Lung Disease, Vol. 5, pp. 703-706. 11495259

[ref054] Sonar, V. (2010), “A retrospective study of prison deaths in Western Maharashtra (2001-2008)”, Medico-Legal Update, Vol. 10.

[ref055] Spittal, M.J., Forsyth, S., Pirkis, J., Alati, R. and Kinner, S.A. (2014), “Suicide in adults released from prison in Queensland, Australia: a cohort study”, Journal of Epidemiology and Community Health, Vol. 68 No. 10, pp. 993-998. 25009152 10.1136/jech-2014-204295

[ref056] Telisinghe, L., Charalambous, S., Topp, S.M., Herce, M.E., Hoffmann, C.J., Barron, P., Schouten, E.J., Jahn, A., Zachariah, R. and Harries, A.D.J.T.L. (2016), “HIV and tuberculosis in prisons in Sub-Saharan Africa”, The Lancet, Vol. 388 No. 10050, pp. 1215-1227. 10.1016/S0140-6736(16)30578-5 PMC6182190 27427448

[ref057] Tirfeneh, E., Abera, M., Yeshigeta, E., Mamaru, A., Dube, L. and Srahbzu, M. (2017), “Suicidal behavior and associated factors among prisoners in Jimma town correctional institution South, west Ethiopia”, Journal of Psychiatry, Vol. 21, p. 451.

[ref058] UN (2018), “Committee against torture examines report of Senegal. United Nations human rights Office - Office of the high commissioner”, available at: www.ohchr.org/en/press-releases/2018/04/committee-against-torture-examines-report-senegal

[ref059] UNODC (2008), Prevention of Spread of HIV Amongst Vulnerable Groups in South Asia, United Nations Office on Drugs and Crime. New Delhi.

[ref060] UNODC (2015), “The United Nations standard minimum rules for the treatment of prisoners (the Nelson Mandela rules)”, United Nations Office on Drugs and Crime, Vienna, Austria.

[ref061] VAN Ginneken, E.F., Sutherland, A. and Molleman, T. (2017), “An ecological analysis of prison overcrowding and suicide rates in England and Wales, 2000–2014”, International Journal of Law and Psychiatry, Vol. 50, pp. 76-82. 27189047 10.1016/j.ijlp.2016.05.005

[ref063] VAN Hout, M.-C. and Mhlanga-Gunda, R. (2019), “Prison health situation and health rights of young people incarcerated in Sub-Saharan African prisons and detention centres: a scoping review of extant literature”, BMC International Health and Human Rights, Vol. 19 No. 1, pp. 1-16. 31118008 10.1186/s12914-019-0200-z PMC6532240

[ref062] VAN Hout, M.C., Mhlanga-Gunda, R. and Rights, H. (2018), “Contemporary women prisoners health experiences, unique prison health care needs and health care outcomes in Sub-Saharan Africa: a scoping review of extant literature”, BMC International Health and Human Rights, Vol. 18 No. 1, pp. 1-12. 30081894 10.1186/s12914-018-0170-6 PMC6080512

[ref064] VAN Hout, M.C., Bigland, C. and Mariniello, T. (2022a), “A legal-realist assessment of the Zimbabwean correctional system response to covid-19 during state disaster measures”, International Journal of Prisoner Health, Vol. 19 No. 3. 10.1108/IJPH-10-2021-0104 35439405

[ref065] VAN Hout, M.C., Mhango, V., Kaima, R., Bigland, C. and Mariniello, T. (2022b), “A legal-realist assessment of human rights, right to health and standards of healthcare in the Malawian prison system during covid-19 state disaster measures”, International Journal of Prisoner Health, Vol. 19 No. 3. 10.1108/IJPH-10-2021-0108 35294830

[ref066] Walmsley, R. (2018), World Prison Population List, 12th ed., International Centre for Prison Studies, Kings College.

[ref067] WHO (1992), “World health organization. International statistical classification of diseases and related health problems (ICD-10)”.

[ref068] WHO (2022), Addressing the Noncommunicable Disease (NCD) Burden in Prisons in the WHO European Region: interventions and Policy Options, World Health Organization. Regional Office for Europe.

[ref069] Winkelman, T.N., Dasrath, K.C., Young, J. and Kinner, S.A. (2022), “Universal health coverage and incarceration”, The Lancet Public Health, Vol. 7 No. 6, pp. e569-e572. 35660218 10.1016/S2468-2667(22)00113-X

[ref070] Wobeser, W.L., Datema, J., Bechard, B. and Ford, P. (2002), “Causes of death among people in custody in Ontario, 1990–1999”, CMAJ: Canadian Medical Association Journal = Journal de L'Association Medicale Canadienne, Vol. 167 No. 10, pp. 1109-1113. PMC134290 12427701

[ref071] WORLDBANK (2023), “Life expectancy at birth, total (years) - Senegal [online]”, available at: https://data.worldbank.org/indicator/SP.DYN.LE00.IN?locations=SN(accessed 19 June 2023).

[ref072] Zhong, S., Senior, M., Yu, R., Perry, A., Hawton, K., Shaw, J. and Fazel, S. (2021), “Risk factors for suicide in prisons: a systematic review and meta-analysis”, The Lancet Public Health, Vol. 6 No. 3, pp. e164-e174. 33577780 10.1016/S2468-2667(20)30233-4 PMC7907684

